# Novel Li_3_VO_4_ Nanostructures Grown in Highly Efficient Microwave Irradiation Strategy and Their In‐Situ Lithium Storage Mechanism

**DOI:** 10.1002/advs.202103493

**Published:** 2021-11-21

**Authors:** Yan Sun, Chunsheng Li, Chen Yang, Guoliang Dai, Lin Li, Zhe Hu, Didi Wang, Yaru Liang, Yuanliang Li, Yunxiao Wang, Yanfei Xu, Yuzhen Zhao, Huakun Liu, Shulei Chou, Zhu Zhu, Miaomiao Wang, Jiahao Zhu

**Affiliations:** ^1^ School of Chemistry and Life Sciences Suzhou University of Science and Technology Suzhou City Jiangsu Province 215009 P.R. China; ^2^ Institute for Carbon Neutralization College of Chemistry and Materials Engineering Wenzhou University Wenzhou Zhejiang 325035 P.R. China; ^3^ Xi'an Key Laboratory of Advanced Photo‐electronics Materials and Energy Conversion Device School of Science Xijing University Xi'an 710123 P.R. China; ^4^ Institute for Superconducting and Electronic Materials University of Wollongong Wollongong NSW 2522 Australia; ^5^ Hebei Provincial Key Laboratory of Inorganic Nonmetallic Materials Key Laboratory of Environment Functional Materials of Tangshan City College of Materials Science and Engineering North China University of Science and Technology Tangshan City Hebei Province 063210 P.R. China

**Keywords:** energy storage mechanism, in‐situ technology, Li_3_VO_4_, microwave irradiation strategy, morphology controlled fabrication

## Abstract

The investigation of novel growth mechanisms for electrodes and the understanding of their in situ energy storage mechanisms remains major challenges in rechargeable lithium‐ion batteries. Herein, a novel mechanism for the growth of high‐purity diversified Li_3_VO_4_ nanostructures (including hollow nanospheres, uniform nanoflowers, dispersed hollow nanocubes, and ultrafine nanowires) has been developed via a microwave irradiation strategy. In situ synchrotron X‐ray diffraction and in situ transmission electron microscope observations are applied to gain deep insight into the intermediate Li_3+_ *_x_* VO_4_ and Li_3+_ *_y_* VO_4_ phases during the lithiation/delithiation mechanism. The first‐principle calculations show that lithium ions migrate into the nanosphere wall rapidly along the (100) plane. Furthermore, the Li_3_VO_4_ hollow nanospheres deliver an outstanding reversible capacity (299.6 mAh g^−1^ after 100 cycles) and excellent cycling stability (a capacity retention of 99.0% after 500 cycles) at 200 mA g^−1^. The unique nanostructure offers a high specific surface area and short diffusion path, leading to fast thermal/kinetic reaction behavior, and preventing undesirable volume expansion during long‐term cycling.

## Graphical abstract

In‐situ transmission electron microscope (TEM) and in situ synchrotron X‐ray diffraction (SXRD) of Li_3_VO_4_ hollow nanospheres observation were applied to gain deep insight into the intermediate Li_3+_ *_x_* VO_4_ and Li_3+_ *_y_* VO_4_ phases during the lithiation/delithiation mechanism.

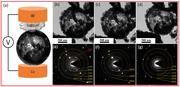

## 1 Introduction

The observation of the highly efficient fabrication mechanism for high‐energy‐density electrode materials in a microwave irradiation field and their in‐situ energy storage mechanism is paramount for advanced rechargeable batteries. In this scheme, however, accurate control of nanostructural growth and the electrochemical reaction mechanism face two major challenges.^[^ ^1^, ^2^, ^3^, ^4^, ^5^, ^6^, ^7^, ^8^, ^9^, ^10^, ^11^, ^12^ ^]^ First, the fast preparation of nanomaterials relies on their thermodynamics (including the most stable state and lowest energy barrier) and kinetics (related to the reaction rate and reaction order) in complicated chemical reactions. As is well known, the electrochemical performances depend strictly on the available electrode materials for rechargeable batteries, such as lithium‐ion batteries (LIBs)^[^ ^13^ ^]^ and sodium‐ion batteries (SIBs). To date, a few vanadium (V)‐based phosphates (e.g., Na_3_V_2_(PO_4_)_2_F_3_,^[^ ^14^ ^]^ Na_3_(VOPO_4_)_2_F,^[^ ^15^ ^]^ Na_3.1_V_2_(PO_4_)_2.9_(SiO_4_)_0.1_,^[^ ^16^ ^]^ etc.) are as cathode applied for SIBs owing to their fast Na^+^ conductivity and high structural stability. Nevertheless, the LIBs are undoubtedly regarded as the most dominant rechargeable batteries over the past three decades,^[^ ^17^ ^]^ because the high energy density^[^ ^18^ ^]^ and long cycle life^[^ ^19^ ^]^ result in various commercial applications of LIBs.^[^ ^20^ ^]^ Second, investigation of the in‐situ storage mechanism requires monitoring the time‐resolved crystalline phase evolution of active material at an atomic scale.^[^ ^21^, ^22^, ^23^, ^24^, ^25^, ^26^ ^]^ Additionally, V‐based materials are indeed important as a type of electrode for LIBs.^[^ ^27^, ^28^ ^]^ Among these anode materials, Li_3_VO_4_ has attracted considerable interest due to its high theoretical capacity (394 mAh g^−1^), high ionic conductivity of Li^+^ through three‐dimensional (3D) pathways in the crystal, safe voltage plateau (0.5‐1.0 V vs. Li/Li^+^), and excellent structural stability for high‐rate capability.^[^ ^29^, ^30^, ^31^, ^32^, ^33^, ^34^ ^]^ Various Li_3_VO_4_ topologies have been fabricated by certain technologies, including solid‐state reaction,^[^ ^35^, ^36^, ^37^, ^38^, ^39^, ^40^, ^41^, ^42^, ^43^, ^44^, ^45^ ^]^ hydro(solvo)thermal route,^[^ ^42^, ^46^, ^47^, ^48^, ^49^, ^50^, ^51^, ^52^, ^53^ ^]^ sol−gel method,^[^ ^54^, ^55^, ^56^, ^57^, ^58^, ^59^, ^60^, ^61^, ^62^, ^63^ ^]^ coordinate electrochemical reconstruction,^[^ ^64^, ^65^ ^]^ freeze‐drying method,^[^ ^66^, ^67^, ^68^ ^]^ the self‐template method,^[^ ^69^ ^]^ ball milling,^[^ ^70^ ^]^ ultrosonic spray pyrolysis,^[^ ^71^, ^72^, ^73^ ^]^ and aerosol‐assisted synthesis.^[^ ^74^ ^]^ To the best of our knowledge, few reports have been focused on the rapid and large‐scale preparation of Li_3_VO_4_ with designed 3D shapes. The high‐efficient microwave strategy remains crucial difficulties arising from the rapid growth of nuclei and the coexistence of stable impurity phases.

Herein, we have created a modified microwave irradiation set‐up with a high revolutionized time relay to monitor the growth mechanism of various pure Li_3_VO_4_ samples, including hollow nanospheres, uniform nanoflowers, dispersed hollow nanocubes, and ultrafine nanowires. Furthermore, the Li‐storage mechanism of these Li_3_VO_4_ nanostructures has been demonstrated via time‐resolved in‐situ synchrotron X‐ray diffraction (SXRD) and in‐situ transmission electron microscope (TEM) technologies to clearly visualize the Li^+^ diffusional pathways and microstructural phase variations in the active materials during electrochemical reactions. Simultaneously, using Li_3_VO_4_ nanospheres as the model, the energy changes of lithium ions entering into different crystal channels from the surface are investigated theoretically. All the results confirmed a novel self‐assembly growth mechanism, dissolution recrystallization, and three‐phase lithiation/delithiation processes for pure Li_3_VO_4_ morphologies, which contribute to the inherent optimization of a promising 3D anode candidate in LIBs.

## 2 Results and Discussion

### 2.1 High‐Efficient Material Fabrications

**Figure 1:**
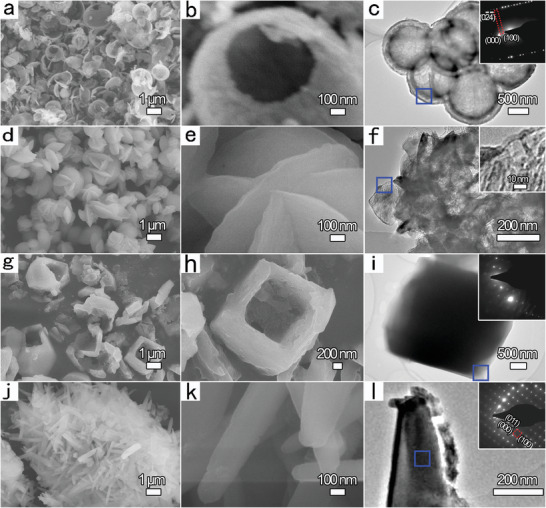
SEM and TEM/HRTEM images of designed Li_3_VO_4_ nanostructures: a−c) hollow nanospheres, d−f) uniform nanoflowers, g−i) dispersed hollow nanocubes, and j−l) ultrafine nanowires. The insets in the HRTEM images show the corresponding experimental SAED patterns of the areas marked by the blue squares in the corresponding HRTEM images, with the exception of the inset to (f), which is a higher magnification image.

Pure Li_3_VO_4_ samples with various nanostructures were designed and synthesized through a modified microwave reactor (Midea PJ21C‐AU with power of 700 W, working program: 20 s on, 10 s off over a frequency of 2450 MHz, Figure S1 in the Supporting Information). Table S1, Supporting Information, summarizes the experimental parameters for altering the raw materials, the concentrations of precursors, and the sorts of surfactants. **Figure** **1**a−c presents the morphology of Li_3_VO_4_ hollow nanospheres (Sample 1) via a hexadecyl trimethylammonium bromide (CTAB)‐assisted fast microwave irradiation process with only 1 min. It can be clearly observed that a large quantity of Li_3_VO_4_ hollow spheres with an extremely uniform diameter of 1.0−1.5 µm is homogeneously dispersed in the wide view in Figure 1a. The high‐magnification scanning electron microscope (SEM) and TEM images presented in Figure 1b−c, respectively, clearly indicate that the thickness of the shells is 80−100 nm. The selected area electron diffraction (SAED) pattern in the inset of Figure 1c supplies a powerful tool to further analyze the detailed surface configuration of Li_3_VO_4_ with orthorhombic phase, which reveals the well‐resolved lattice fringes of the ($0\bar{2}\bar{4}$) and (100) crystal planes. The SAED pattern indicates the single‐crystalline nature of the product, which is consistent with the X‐ray diffraction (XRD) pattern (Figure S2 in the Supporting Information). After changing the concentrations of precursors (Table S1 in the Supporting Information), Li_3_VO_4_ nanoflowers (Sample 2) were generated by microwave irradiation for 1 h. The surface morphology and structure of the product were confirmed by applying SEM and TEM/high‐resolution TEM (HRTEM) (Figure 1d−f). A great number of 3D flower‐like Li_3_VO_4_ hierarchical nanostructures are composed of ordered nanosheets with an average diameter of 1.0−2.1 µm and thickness ranging from 25 to 50 nm. Surprisingly, after the addition of ethylenediaminetetraacetic acid (EDTA) in the microwave irradiation process, monodispersed hollow Li_3_VO_4_ nanocubes (Sample 3) were controllably generated over the entire area for the first time (Figure 1g−i and Table S1, Supporting Information). The cube‐shaped hollow product is highly uniform, with each cube having a side length of 2.0−3.0 µm with sharp corners (Figure 1g), whereas the wall thickness is approximately 90−150 nm (Figure 1h). Furthermore, the SAED pattern of Li_3_VO_4_ hollow nanocubes (inset to the TEM image in Figure 1i) clearly reveals the single‐crystalline nature of the product with the diffraction spots well indexed to the orthorhombic Li_3_VO_4_ phase. On using the combination of the solid‐state method and the microwave irradiation route, abundant Li_3_VO_4_ nanowires with diameters of 80−240 nm were found to dominate the prevailing morphology (Sample 4, Figure 1j−k). Moreover, the length of nanowires is up to 3 µm, as shown in the SEM images. HRTEM investigation reveals the lattice defects of the individual nanowires, suggesting a single‐crystalline texture. In addition, the diffraction pattern in the SAED pattern (Figure 1l) obviously indicates clear reflection spots that can be attributed to the (011) and (100) lattice planes, verifying the single crystallinity of the Li_3_VO_4_ nanowires along the [100] growth direction.

**Figure 2:**
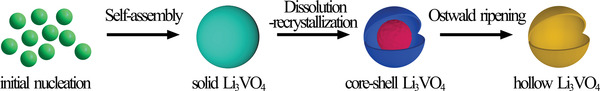
The schematic illustration of the growth mechanism of “self‐assemble‐dissolution recrystallization” for Li_3_VO_4_ hollow spheres.

To monitor the growth mechanism of the above Li_3_VO_4_ nanostructures, the morphology and phase changes of samples over the reaction time were systematically probed by SEM and XRD (**Figure** **2**, Figure S3−S10 and Tables S2−S5, Supporting Information). Therefore, a precise “self‐assemble‐dissolution recrystallization” mechanism can explain the formation processes of the unique Li_3_VO_4_ morphologies (hollow nanospheres, uniform nanoflowers, dispersed hollow nanocubes, and ultrafine nanowires). Up to now, the growth mechanism represents an obvious innovation for the highly efficient microwave irradiation preparation of Li_3_VO_4_ nanostructures (Figure 2) and is quite different from those in the published works,^[^ ^46^, ^49^, ^51^, ^52^, ^59^, ^64^, ^66^, ^69^, ^74^ ^]^ but the mechanism has generally appeared in the rapid synthesis of ZnV_2_O_6_ and Ba_2_V_2_O_7_ nanomaterials in our previous studies.^[^ ^75^, ^76^, ^77^ ^]^

### 2.2 The Exploration of Energy Storage Mechanism

#### 2.2.1 In‐Situ Synchrotron Diffraction Characterization

**Figure 3:**
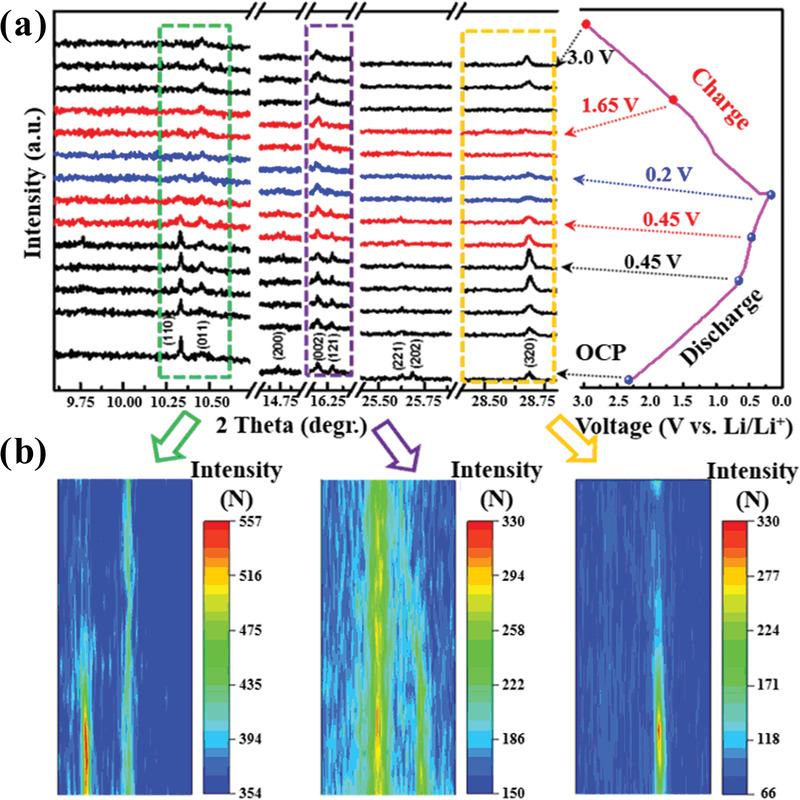
a) Selected regions (left) of in‐situ SXRD patterns of Li_3_VO_4_ hollow nanospheres (Sample 1) at representative charge/discharge states during the first cycle at a current density of 100 mA g^−1^, and the corresponding voltage‐profile (right) in‐situ measurement. b) Contour plots of SXRD patterns in the denoted ranges of degrees.

In‐situ synchrotron diffraction technology, as an excellent analytical tool, can directly identify the real‐time structural variations of electrode nanomaterials during charge−discharge cycling,^[^ ^3^, ^4^, ^5^ ^]^ which is critical for understanding the Li‐storage mechanism and relative structural changes in the electrode. High‐resolution in‐situ SXRD patterns were collected via the Powder Diffraction Beamline at the Australian Synchrotron. **Figure** **3** illustrates the in‐situ SXRD patterns of a half‐cell with Li_3_VO_4_ hollow nanospheres (Sample 1) as an anode with corresponding charge and discharge curves. When the cell is discharged to 0.65 V, an intermediate is formed, which shows overlapped phases with Li_3_VO_4_ at the (002) and (320) Bragg reflections, along with three new reflections that emerged at 9.70°, 14.79° and 25.63°. When discharged to 0.45 V, with the disappearance of more peaks, a Li_3+_ *_x_* VO_4_ phase that is formed has similar peaks to Li_3_VO_4_ at the observed (002), (211), (013), and (213) reflections, corresponding to Li insertion (Equation (1)). Meanwhile, the dominant peaks at (110) and (011) completely disappear, indicating that Li_3_VO_4_ has vanished:

(1) $$Li_{3}VO_{4}+x\mathrm{Li}\to Li_{3+x}VO_{4}\;\left(0<x<1.5\right)$$

At the discharged state at 0.20 V, Li_3+_ *_x_* VO_4_ can be converted into a new phase with reflections at 10.38° and 16.25°, indicating that (Li_3+_ *_y_* VO_4_) has been produced. Only the (002) peak and a very small (213) reflection are retained, which is likely due to the overlapping peaks with Li_3+_ *_x_* VO_4_ (see Equation (2)):

(2) $$Li_{3+x}VO_{4}+\left(y-x\right)\mathrm{Li}\to Li_{3+y}VO_{4}\;\left(1.5\leq y<2\right)$$

As shown in Figure 3b, when fully charged back to 3 V, the reversible peaks of the (011), (002), and (320) reflections of Li_3_VO_4_ are recovered, which confirm the reversible insertion/de‐insertion mechanism. Interestingly, the (011) and (002) peaks show a little variation during the charge/discharge process, probably because it is the overlapping peak for Li_3_VO_4_, Li_3+_ *_x_* VO_4_ and Li_3+_ *_y_* VO_4_. The overlapping peaks of these three compounds indicate a similar crystalline structure, indicating the low volume expansion of Li_3_VO_4_ for Li‐ion storage. Even when combining these results with previous reports, it is still hard to determine the structural details of the new phases Li_3+_ *_x_* VO_4_ and Li_3+_ *_y_* VO_4_, but this is the convincing evidence that Li_3_VO_4_ undergoes a reversible insertion/deinsertion mechanism during the discharge/charge process.

#### 2.2.2 In‐Situ TEM Characterization of Electrochemical Performances

**Figure 4:**
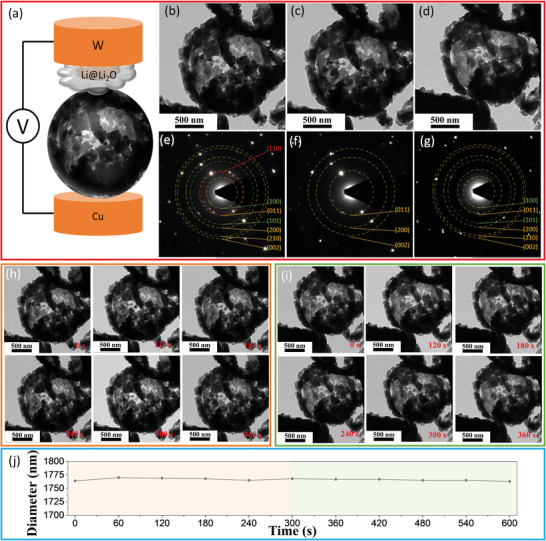
a) Schematic of the experimental setup used for in‐situ TEM measurements, TEM images of the Li_3_VO_4_ hollow nanosphere b) before lithiation, c) after lithiation, d) after delithiation, e−g) SAED patterns of the corresponding stages, time lapse TEM images of h) the lithiation and i) delithiation, and j) Variation of the structural parameters.

To further understand the Li^+^ insertion/desertion processes of the Li_3_VO_4_, the real‐time lithiation of the Li_3_VO_4_ hollow nanosphere was observed using in‐situ TEM. With an in‐situ holder, a nanoscale battery system (half‐cell), illustrated as **Figure** **4**a consisted of the hollow nanosphere as the working electrode and Li metal covered by a natural thin Li_2_O solid electrolyte layer as the counter electrode. Changes in the morphology and phase of a single Li_3_VO_4_ hollow nanosphere before and after the (de)lithation process are shown in Figure 4. The dynamic structural evolution can be found in the Supporting Information, Movie S1. Figure 4b shows the morphology of the Li_3_VO_4_ hollow nanosphere, which is in contact with Li_2_O solid electrolyte before lithiation. Figure 4e reveals the corresponding SAED pattern of the hollow nanosphere with the diffraction dots of (100), (110), (011), (101), (200), (210), and (002) planes of Li_3_VO_4_. No obvious volume expansion of the Li_3_VO_4_ hollow nanosphere occurred after a bias voltage of −3 V applied (Figure 4h), indicating the hollow nanosphere structure is stable to lithiation. After full lithiation, a small volume expansion of about 0.22% is measured. To understand the microstructure of the Li_3_VO_4_ after lithiation, the SAED pattern was taken from it. The SAED pattern as shown in Figure 4f indicates that, after the first discharging process, the pristine Li_3_VO_4_ becomes lithiated and transformed to a new phase, with the disappeared (001), (011), (101), and (210) planes, which is consistent with the phenomenon in the in‐situ SXRD data.

The lithiated hollow nanosphere was charged again by reversing the bias to understand the delithiation process. Figure 3d reveals the morphology of the same hollow nanosphere after full delithiation. It should be noted that there is a negligible decrease in the nanosphere diameter. The corresponding SAED analysis indicates that the phase of delithiated hollow nanosphere is back to Li_3_VO_4_ as revealed in Figure 4g. The Li_3_VO_4_ hollow nanosphere is found to undergo reversible changes during the cycling process (Figure 4i), and the direct in‐situ observation shown here indicates that the reversible changes can be responsible for the good cycling stability of Li_3_VO_4_ as anode material for LIBs.

### 2.3 Theoretical Calculations of Li_3_VO_4_ Crystal Planes

**Figure 5:**
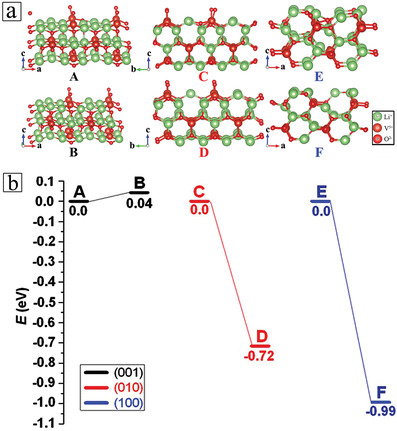
a) The optimized structures of Li_3_VO_4_. The green and red (big) spheres represent the Li and V atoms, respectively, while the red (small) sphere represents the O atom. Model A, C, and E indicate the defects on the surface of (001), (010), and (100), respectively. Model B, D and F illustrates the migration of lithium ions from the surface to bulk phase of (001), (010), and (100) surfaces, respectively. b) Schematic energy profile corresponding to local configurations shown in Figure 5a.

To elucidate the lithium‐ion insertion mechanism in the Li_3_VO_4_ hollow nanospheres, the first‐principle calculations were performed (see methods for details) on different plane surfaces in a supercell of Li_3_VO_4_. We designed a lithium‐ion hole in the pure crystal phase of the sphere wall (model A, C, and E in **Figure** **5**a represent the defects in the material on the surface of (001), (010), and (100) planes, respectively), and calculated the energy change of the surface lithium‐ion entering into the body phase to fill the empty holes (model B, D, and F in Figure 5a illustrate the migration of lithium ions from surface to bulk phase in (001), (010), and (100) surfaces, respectively). Obviously, during the migration of lithium ions from the surface layer to the interior of the bulk phase through the wall of hollow nanospheres over the (001) surface, there is little change in energy, and only 0.04 eV of energy is required. On the (010) and (100) surfaces, the lithium‐ion migration into the bulk phase of hollow nanospheres is a spontaneous exothermic process, which releases 0.72 and 0.99 eV energy, respectively. It is clear that, the lithium‐ion can migrate easily into the Li_3_VO_4_ hollow nanospheres during charging/discharging along the (100) plane.

### 2.4 Electrochemical Characterization

**Figure 6:**
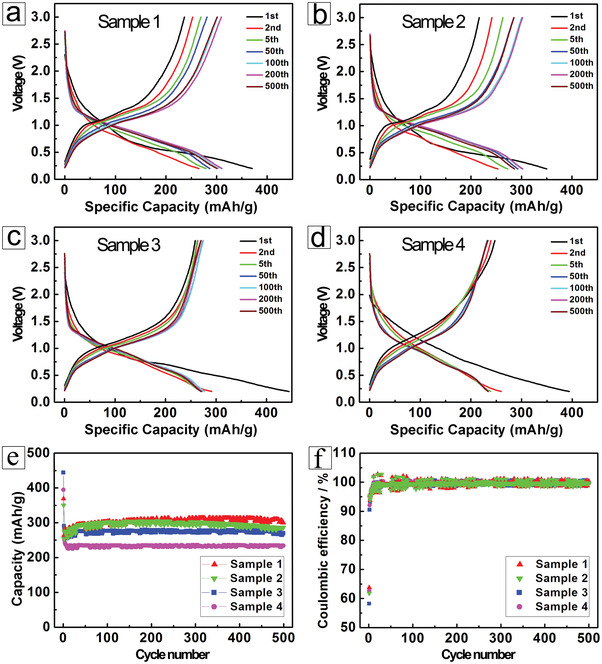
Galvanostatic charge−discharge profiles for selected cycles between 3.00 and 0.20 V versus Li/Li^+^ at a constant current density of 200 mA g^−1^ in LIBs: a) Sample 1, hollow nanospheres, b) Sample 2, uniform nanoflowers, c) Sample 3, dispersed hollow nanocubes, and d) Sample 4, ultrafine nanowires. e) Cycling properties of these electrodes assembled from these Li_3_VO_4_ nanostructures. f) Coulombic efficiency as a function of cycle number for Samples 1−4.

Benefit from the high surface area and ultrathin Li_3_VO_4_ walls in the nanostructures, all of the pure Li_3_VO_4_ nanostructures could realize intimate contact with electrolytes and shorten Li^+^ diffusion paths, thereby improving their Li‐storage properties. The electrochemical properties of the novel Li_3_VO_4_ nanomaterials in the potential window ranging from 0.20 to 3.00 V versus Li/Li^+^ were systematically investigated, and the results are shown in **Figure** **6** and Figure S11, Supporting Information. As shown in Figure 6a−d, the first‐insertion capacities are 368.9, 350.0, 443.8, and 394.4 mAh g^−1^ for Li_3_VO_4_ hollow nanospheres (Sample 1), uniform nanoflowers (Sample 2), dispersed hollow nanocubes (Sample 3), and ultrafine nanowires (Sample 4), respectively. The corresponding capacities at the second discharge cycle are decreased, however, to 264.0, 253.4, 290.9, and 260.0 mAh g^−1^. The remarkable irreversible capacity fading between the initial and subsequent cycles demonstrates an intrinsic characteristic of the materials, which can be attributed to the formation of a solid electrolyte interphase film and the initial irreversible lithiation reaction.^[^ ^70^, ^74^, ^78^, ^79^, ^80^ ^]^ Indeed, the charge−discharge performance fully stabilizes after 100 cycles. As shown in Figure 6a−b, it is impressive that Sample 1 and Sample 2 exhibit the highest specific capacities of ≈305 mAh g^−1^ over the initial 150 cycles. During the following 300 cycles, Sample 1 maintains excellent stability with a capacity of ≈300 mAh g^−1^. For the following 200 cycles, the electrode made from Li_3_VO_4_ hollow nanospheres shows increased capacity up to 310.8 mAh g^−1^. A lower capacity of ≈300 mAh g^−1^ could be maintained beyond 500 cycles. Additionally, it is clear that the intercalation reaction of Li_3_VO_4_ mainly occurs in the voltage range from 1.0 to 0.5 V, which favorably avoids the deposition of lithium dendrites, endowing this anode with higher safety.^[^ ^40^ ^]^ In agreement with the in‐situ SXRD results, the Li_3+_ *_x_* VO_4_ phase is proposed to be formed during this process. When further discharged to 0.2 V, sloping and shortened discharge curves are observed, corresponding to the Li_3+_ *_y_* VO_4_ phase. During the charging process, flat charge plateaus appear from 1.0−1.5 V, which corresponds to the reversible reaction of Li^+^ extraction from Li_3+_ *_y_* VO_4_ to Li_3_VO_4_ phase.

In addition, the long‐term cycling stability of the as‐obtained Li_3_VO_4_ electrodes up to 500 cycles (Figure 6e−f), illustrates their highly reversible energy storage and conversion, and capacity of 299.6, 286.5, 269.8, and 233.9 mAh g^−1^ is still achieved, respectively. Obviously, the capacitance retention of Sample 1−4 can still be maintained around 99.0%, 94.8%, 97.7%, and 99.8% even after cycling 500 times (versus the 100^th^ cycle), implying the outstanding electrochemical activity towards Li^+^ insertion/extraction. Strikingly, the specific capacities of the Li_3_VO_4_ materials in order are Sample 1 > Sample 2 > Sample 3 > Sample 4, agreeing well with their particle size and structural stability. It is also worth mentioning that the superior properties of the Li_3_VO_4_ hollow spheres (Sample 1) with the superior electrochemical properties are plausibly assigned to the following reasons. To begin with, the novel hollow spheres not only possess ultrathin walls approximately 80−100 nm in thickness, which enlarges the specific surface area, but also improves the rapid mass transfer of lithium ions to more active sites during the electrode reactions. What is more, compared with other Li_3_VO_4_ morphologies in this study, the unique hollow sphere structure undoubtedly prevents undesirable volume expansion during cell cycling and thus ensures excellent structural stability, which is critical to avoid capacity decay even at high current density (200 mA g^−1^) over long‐term cycling (500 cycles). Therefore, these merits will convincingly pave the way to promising applications in practical LIBs with fast reaction thermal/kinetic behavior, high energy density, and favorable cycling stability.

## 3 Conclusion

In summary, a novel growth mechanism based on a rapid microwave irradiation strategy for high‐purity Li_3_VO_4_ hollow nanospheres, uniform nanoflowers, dispersed hollow nanocubes, and ultrafine nanowires has been systematically investigated. Moreover, to ascertain the Li‐storage mechanism of Li_3_VO_4_, in‐situ SXRD was performed at various charge/discharge states. The obtained data unambiguously proved that two reversible intermediate phases, Li_3+_ *_x_* VO_4_ (0<*x*<1.5) and Li_3+_ *_y_* VO_4_ (1.5<y<2), were formed during the discharge at 0.45 V and 0.20 V, respectively. When charged back to 3.00 V, the reversible peaks were dominated by the initial Li_3_VO_4_ phase with the dramatic reduction of the Li_3+_ *_x_* VO_4_ and Li_3+_ *_y_* VO_4_ phases in the charging process. These results are in good agreement with our first‐principle calculation, through which we find that lithium‐ion can enter into the inner layer of the material over (100) surface easiest. It directly shows the evidence of the reversible insertion/deinsertion mechanism of Li_3_VO_4_ nanostructure. Benefiting from their unique electrode structure, Li_3_VO_4_ hollow nanospheres exhibited an excellent initial specific capacity (368.9 mAh g^−1^), superior reversible capacity at the 100^th^ cycle (299.6 mAh g^−1^), and outstanding cycling stability (around 99.0% retention even over 500 cycles at the high current density of 200 mA g^−1^). Therefore, these encouraging advantages endowed the Li_3_VO_4_ 3D nanomaterials with great promise as a candidate for advanced usage in energy storage devices.

## 4 Experimental Section

### Materials Characterization

The microstructure of the as‐prepared Li_3_VO_4_ nanomaterials was performed on XRD (Rigaku D/MAX2500PC diffractometer using Cu K*α* with *λ* = 1.54056 Å), field‐emission scanning electron microscopy (FE‐SEM, Hitachi S‐4800 using an accelerating voltage of 10 kV), and transmission electron microscope (TEM)/high‐resolution TEM (HRTEM) (a Philips Tecnai G2 F20 at acceleration voltage of 200 kV).

### Electrochemical Measurements

The electrochemical tests were conducted by assembling coin‐type CR2023 cells in an argon‐filled glove box. The slurry consisted of 80 wt.% Li_3_VO_4_, 10 wt.% carbon black, and 10 wt.% polyvinylidene fluoride (PVDF). The Li_3_VO_4_ electrode can be obtained by pasting the slurry on copper foil using a doctor blade with a thickness of 100 µm, which was followed by drying at 120 °C in a vacuum oven overnight. The working electrodes were prepared by punching the electrode film into discs 0.96 cm in diameter. Lithium foil was employed for both reference and counter electrodes. The electrodes were separated by a Celgard separator. The electrolyte was 1.0 M LiPF_6_ in 3:4:3 (weight ratio) of ethylene carbonate (EC)/dimethyl carbonate (DMC)/diethylene carbonate (DEC), with a 5 wt.% fluoroethylene carbonate (FEC) additive from Novolyte Technologies. The electrochemical performances were tested on a Land battery test system with a cut‐off voltage range from 0.20 to 3.00 V (vs. Li /Li^+^). Cyclic voltammetry and impedance testing were performed using a Biologic VMP‐3 electrochemical workstation from 0.20 to 3.00 V at a sweep rate of 0.05 mV s^−1^.

### In‐Situ Synchrotron XRD Measurements

The cells for in‐situ synchrotron XRD were similar to the above‐mentioned coin‐cells for electrochemical performance. To enhance the intensity of diffraction peaks, much thick cathode materials were loaded onto the Cu foil, which was up to 5 mg cm^−2^. To collect the signals from the full cell, two holes with a diameter of 4 mm were punched on the negative and positive caps, respectively, guaranteeing that the X‐ray beams could go through the whole cell and monitor the electrochemical reaction. Afterward, the holes of the negative and positive caps were covered by Kapton film (only showing one slight bump in XRD measurements), followed by completely sealed with AB glue. Meanwhile, a battery test system (Neware) was connected to carry out the charge/discharge process.

### In‐Situ TEM Measurements

In‐situ TEM was conducted using a TF20 transmission electron microscope.^[^ ^75^, ^76^ ^]^ A nanoscale electrochemical cell was assembled using a TEM‐STEM holder (Pico Femto FE02‐ST) obtained from Zeptools Co., Ltd. A copper grid loaded with Li_3_VO_4_ hollow nanospheres (sample 1) was set as the anode, and a tungsten probe coated with lithium was set as the cathode. The lithium was exposed to air for about 5−10 s to form a thin layer of lithium oxide, which can act as a solid electrolyte. To begin the in‐situ TEM experiment, the lithium oxide was positioned to touch the sample of Li_3_VO_4_ hollow nanospheres, and a bias voltage of −3 V was applied to enable Li^+^ diffusion through the Li_2_O layer. In delithiation processes, a bias voltage of 3 V was applied.

### Theoretical Calculations

The first‐principles calculations were performed using the Vienna ab initio software package (VASP). The generalized gradient approximation (GGA) with the Perdew–Burke–Ernzerhof (PBE) parameterization was employed as the electron exchange‐correlation functional. The projector augmented‐wave (PAW) method was used to describe the wave functions near the core. The cell shape volume and atomic positions have been fully optimized until the remaining forces are lower than 0.01 eV Å^–1^. Three planes (001), (100), and (010) were considered in the present calculations. A (2×2) unit cell was used to model the surface and the vacuum region was 20 Å. The 3×3×1 grid was employed for K‐space sampling. Spin‐polarization was considered during all the calculations.

## Conflict of Interest

The authors declare no conflict of interest.

## Supporting information

Supporting Information

Supplemental Video 1

## Data Availability

Research data are not shared.

## References

[1] R. Boston , Z. Schnepp , Y. Nemoto , Y. Sakka , S. R. Hall , Science 2014, 344, 623. 24812400 10.1126/science.1251594

[2] J. Y. Huang , L. Zhong , C. M. Wang , J. P. Sullivan , W. Xu , L. Q. Zhang , S. X. Mao , N. S. Hudak , X. H. Liu , A. Subramanian , H. Fan , L. Qi , A. Kushima , J. Li , Science 2010, 330, 1515. 21148385 10.1126/science.1195628

[3] G. D. Du , N. Sharma , V. K. Peterson , J. A. Kimpton , D. Z. Jia , Z. P. Guo , Adv. Funct. Mater. 2011, 21, 3990.

[4] E. W. Zhao , E. Jónsson , R. B. Jethwa , D. Hey , D. X. Lyu , A. Brookfield , P. A. A. Klusener , D. Collison , C. P. Grey , J. Am. Chem. Soc. 2021, 143, 1885. 33475344 10.1021/jacs.0c10650 PMC7877726

[5] H. F. Qiu , J. Wan , J. X. Zhang , X. Wang , N. J. Zhang , R. X. Chen , Y. Xia , L. Huang , H. L. Wang , Adv. Sci. 2021, 2101759. 10.1002/advs.202101759 PMC8425916 34250756

[6] Z. Cui , S. A. He , Q. Liu , G. Q. Guan , W. L. Zhang , C. T. Xu , J. Q. Zhu , P. Feng , J. Q. Hu , R. J. Zou , M. F. Zhu , Adv. Sci. 2020, 7, 1903045. 10.1002/advs.201903045 PMC7509643 32999824

[7] Z. G. Yang , Z. G. Wu , W. B. Hua , Y. Xiao , G. K. Wang , Y. X. Liu , C. J. Wu , Y. C. Li , B. H. Zhong , W. Xiang , Y. J. Zhong , X. D. Guo , Adv. Sci. 2020, 7, 1903279. 10.1002/advs.201903279 PMC7284207 32537402

[8] X. L. Dong , Y. Yang , B. L. Wang , Y. J. Cao , N. Wang , P. L. Li , Y. G. Wang , Y. Y. Xia , Adv. Sci. 2020, 7, 2000196. 10.1002/advs.202000196 PMC7375234 32714749

[9] Y. F. Yuan , K. Amine , J. Lu , R. Shahbazian‐Yassar , Nat. Commun. 2017, 8, 15806.

[10] C. Y. Fan , X. H. Zhang , Y. H. Shi , H. Y. Xu , J. P. Zhang , X. L. Wu , J. Mater. Chem. A 2019, 7, 1529.

[11] J. C. Zhang , S. B. Ni , T. Kang , J. Tang , X. L. Yang , L. L. Zhang , J. Mater. Chem. A 2016, 4, 14101.

[12] H. T. Huu , N. H. Vu , H. Ha , J. Moon , H. Y. Kim , W. Bin Im , Nat. Commun. 2021, 12, 3081. 34035270 10.1038/s41467-021-23366-8 PMC8149873

[13] Q. Ni , L. M. Zheng , Y. Bai , T. C. Liu , H. X. Ren , H. J. Xu , C. Wu , J. Lu , ACS Energy Lett. 2020, 5, 1763.

[14] Z. Y. Gu , J. Z. Guo , X. X. Zhao , X. T. Wang , D. Xie , Z. H. Sun , C. D. Zhao , H. J. Liang , W. H. Li , X. L. Wu , InfoMat 2021, 3, 694.

[15] B. H. Deng , N. Yue , H. Y. Dong , Q. Y. Gui , L. Xiao , J. P. Liu , Chin. Chem. Lett. 2021, 32, 826.

[16] M. Y. Wang , J. Z. Guo , Z. W. Wang , Z. Y. Gu , X. J. Nie , X. Yang , X. L. Wu , Small 2020, 16, 1907645. 10.1002/smll.201907645 32141157

[17] F. Y. Cheng , J. Liang , Z. L. Tao , J. Chen , Adv. Mater. 2011, 23, 1695. 21394791 10.1002/adma.201003587

[18] M. T. McDowell , S. W. Lee , W. D. Nix , Y. Cui , Adv. Mater. 2013, 25, 4966. 24038172 10.1002/adma.201301795

[19] Q. H. Wang , J. T. Xu , W. C. Zhang , M. L. Mao , Z. X. Wei , L. Wang , C. Y. Cui , Y. X. Zhu , J. M. Ma , J. Mater. Chem. A 2018, 6, 8815.

[20] X. B. Meng , X. Q. Yang , X. L. Sun , Adv. Mater. 2012, 24, 3589. 22700328 10.1002/adma.201200397

[21] K. C. Huang , H. H. Li , H. H. Fan , J. Z. Guo , Y. M. Xing , Y. P. Hu , X. L. Wu , J. P. Zhang , ChemElectroChem 2017, 4, 2293.

[22] J. Z. Guo , P. F. Wang , X. L. Wu , X. H. Zhang , Q. Y. Yan , H. Chen , J. P. Zhang , Y. G. Guo , Adv. Mater. 2017, 29, 1701968.

[23] S. B. Ni , J. L. Liu , D. L. Chao , L. Q. Mai , Adv. Energy Mater. 2019, 9, 1803324.

[24] R. Asakura , C. Bolli , P. Novák , R. Robert , ChemElectroChem 2020, 7, 2033.

[25] L. L. Zhou , S. Y. Shen , X. Peng , L. N. Wu , Q. Wang , C. H. Shen , T. T. Tu , L. Huang , J. T. Li , S. G. Sun , ACS Appl. Mater. Interfaces 2016, 8, 23739. 27556414 10.1021/acsami.6b07811

[26] X. Jin , B. B. Lei , J. Wang , Z. L. Chen , K. Xie , F. L. Wu , Y. Song , D. L. Sun , F. Fang , J. Alloys Compd. 2016, 686, 227.

[27] M. M. Wang , C. S. Li , Y. Sun , C. Yang , L. Li , Z. Zhu , D. D. Wang , J. H. Zhu , Y. L. Li , S. L. Chou , J. Mater. Chem. C 2021, 10.1039/D1TC03757A.

[28] Y. Yang , J. Q. Li , J. X. Huang , J. X. Huang , J. Zeng , J. B. Zhao , Electrochim. Acta 2017, 247, 771.

[29] S. Li , Z. T. Qu , S. Luo , Y. Guo , Z. M. Wan , X. Z. Kong , ChemElectroChem 2020, 7, 3984.

[30] C. N. Mu , K. X. Lei , H. X. Li , F. J. Li , J. Chen , J. Phys. Chem. C 2017, 121, 26196.

[31] J. Mo , X. M. Zhang , J. J. Liu , J. G. Yu , Z. A. Wang , Z. C. Liu , X. H. Yuan , C. J. Zhou , R. L. Li , X. W. Wu , Y. P. Wu , Chin. J. Chem. 2017, 35, 1789.

[32] J. F. Zhou , B. C. Zhao , J. Y. Song , B. Z. Chen , J. Bai , Z. T. Fang , J. M. Dai , X. B. Zhu , Y. P. Sun , ACS Appl. Energy Mater. 2019, 2, 354.

[33] C. Y. Liao , Y. W. Wen , Z. G. Xia , R. H. Qin , X. Liu , Y. Yu , B. Shan , T. Y. Zhai , H. Q. Li , Adv. Energy Mater. 2018, 8, 1701621.

[34] Z. Q. Yan , Z. H. Sun , A. Q. Xia , R. Yin , X. S. Huang , K. C. Yue , H. R. Xu , G. J. Zhao , L. Qian , Ceram. Int. 2020, 46, 2247.

[35] B. Dong , R. Jarkaneh , S. Hull , N. Reeves‐McLaren , J. J. Biendicho , A. R. West , J. Mater. Chem. A 2016, 4, 1408.

[36] Q. D. Li , Q. L. Wei , J. Z. Sheng , M. Y. Yan , L. Zhou , W. Luo , R. M. Sun , L. Q. Mai , Adv. Sci. 2015, 2, 1500284. 10.1002/advs.201500284 PMC5054844 27774378

[37] K. Huang , Q. N. Ling , C. H. Huang , K. Bi , W. J. Wang , T. Z. Yang , Y. K. Lu , J. Liu , R. Zhang , D. Y. Fan , Y. G. Wang , M. Lei , J. Alloys Compd. 2015, 646, 837.

[38] L. Chen , X. L. Jiang , N. N. Wang , J. Yue , Y. T. Qian , J. Yang , Adv. Sci. 2015, 2, 1500090. 10.1002/advs.201500090 PMC5033021 27709001

[39] C. K. Zhang , H. Q. Song , C. F. Liu , Y. J. Liu , C. P. Zhang , X. H. Nan , G. Z. Cao , Adv. Funct. Mater 2015, 25, 3497.

[40] Z. Y. Liang , Y. M. Zhao , Y. Z. Dong , Q. Kuang , X. H. Lin , X. D. Liu , D. L. Yan , J. Electroanal. Chem. 2015, 745, 1.

[41] Z. Y. Liang , Z. P. Lin , Y. M. Zhao , Y. Z. Dong , Q. Kuang , X. H. Lin , X. D. Liu , D. L. Yan , J. Power Sources 2015, 274, 345.

[42] S. B. Ni , J. C. Zhang , J. J. Ma , X. L. Yang , L. L. Zhang , J. Power Sources 2015, 296, 377.

[43] H. Q. Li , X. Z. Liu , T. Y. Zhai , D. Li , H. S. Zhou , Adv. Energy Mater. 2013, 3, 428.

[44] C. Y. Liao , Y. W. Wen , B. Shan , T. Y. Zhai , H. Q. Li , J. Power Sources 2017, 348, 48.

[45] X. T. Wang , B. Qin , D. Sui , Z. H. Sun , Y. Zhou , H. T. Zhang , Y. S. Chen , Energy Technol. 2018, 6, 2074.

[46] P. F. Zhang , L. Z. Zhao , Q. Y. An , Q. L. Wei , L. Zhou , X. J. Wei , J. Z. Sheng , L. Q. Mai , Small 2016, 12, 1082. 26726814 10.1002/smll.201503214

[47] J. Liu , P. J. Lu , S. Q. Liang , J. Liu , W. J. Wang , M. Lei , S. S. Tang , Q. Yang , Nano Energy 2015, 12, 709.

[48] S. B. Ni , X. H. Lv , J. J. Ma , X. L. Yang , L. L. Zhang , Electrochim. Acta 2014, 130, 800.

[49] Y. Shi , J. Gao , H. D. Abruña , H. J. Li , H. K. Liu , D. Wexler , J. Z. Wang , Y. P. Wu , Chem. Eur. J. 2014, 20, 5608. 24687863 10.1002/chem.201400118

[50] S. B. Ni , X. H. Lv , J. J. Ma , X. L. Yang , L. L. Zhang , J. Power Sources 2014, 248, 122.

[51] Q. D. Li , J. Z. Sheng , Q. L. Wei , Q. Y. An , X. J. Wei , P. F. Zhang , L. Q. Mai , Nanoscale 2014, 6, 11072. 25155363 10.1039/c4nr03119a

[52] Y. Shi , J. Z. Wang , S. L. Chou , D. Wexler , H. J. Li , K. Ozawa , H. K. Liu , Y. P. Wu , Nano Lett. 2013, 13, 4715. 24024651 10.1021/nl402237u

[53] L. F. Shen , H. F. Lv , S. Q. Chen , P. Kopold , P. A. van Aken , X. J. Wu , J. Maier , Y. Yu , Adv. Mater. 2017, 29, 1700142. 10.1002/adma.201700142 28466539

[54] S. Hu , Y. F. Song , S. Y. Yuan , H. M. Liu , Q. J. Xu , Y. G. Wang , C. X. Wang , Y. Y. Xia , J. Power Sources 2016, 303, 333.

[55] C. K. Zhang , C. F. Liu , X. H. Nan , H. Q. Song , Y. J. Liu , C. P. Zhang , G. Z. Cao , ACS Appl. Mater. Interfaces 2016, 8, 680. 26653537 10.1021/acsami.5b09810

[56] J. C. Zhang , S. B. Ni , J. J. Ma , X. L. Yang , L. L. Zhang , J. Power Sources 2016, 301, 41.

[57] C. Q. Du , J. W. Wu , J. Liu , M. Yang , Q. Xu , Z. Y. Tang , X. H. Zhang , Electrochim. Acta 2015, 152, 473.

[58] H. Y. Wei , D. S. Tsai , C. L. Hsieh , RSC Adv. 2015, 5, 69176.

[59] Z. L. Jian , M. B. Zheng , Y. L. Liang , X. X. Zhang , S. Gheytani , Y. C. Lan , Y. Shi , Y. Yao , Chem. Commun. 2015, 51, 229. 10.1039/c4cc07444k 25406736

[60] S. B. Ni , X. H. Lv , J. C. Zhang , J. J. Ma , X. L. Yang , L. L. Zhang , Electrochim. Acta 2014, 145, 327.

[61] Z. Y. Liang , Y. M. Zhao , L. Z. Ouyang , Y. Z. Dong , Q. Kuang , X. H. Lin , X. D. Liu , D. L. Yan , J. Power Sources 2014, 252, 244.

[62] W. T. Kim , Y. U. Jeong , Y. J. Lee , Y. J. Kim , J. H. Song , J. Power Sources 2013, 244, 557.

[63] I. Mulaudzi , Y. Zhang , G. F. Ndlovu , Y. P. Wu , M. A. Legodi , T. van Ree , Electroanalysis 2020, 32, 2635.

[64] S. B. Ni , J. C. Zhang , J. J. Ma , X. L. Yang , L. L. Zhang , X. M. Li , H. B. Zeng , Adv. Mater. Interfaces 2016, 3, 1500340.

[65] S. B. Ni , J. C. Zhang , X. H. Lv , X. L. Yang , L. L. Zhang , J. Power Sources 2015, 291, 95.

[66] D. Zhao , M. H. Cao , ACS Appl. Mater. Interfaces 2015, 7, 25084. 26502345 10.1021/acsami.5b05269

[67] K. Wang , C. K. Zhang , H. Y. Fu , C. F. Liu , Z. Y. Li , W. D. Ma , X. M. Lu , G. Z. Cao , Chem. Eur. J. 2017, 23, 5368. 28244211 10.1002/chem.201700150

[68] X. Q. Liu , G. S. Li , D. Zhang , D. D. Chen , X. Y. Wang , B. Y. Li , L. P. Li , Electrochim. Acta 2019, 308, 185.

[69] Q. D. Li , Q. L. Wei , Q. Q. Wang , W. Luo , Q. Y. An , Y. N. Xu , C. J. Niu , C. J. Tang , L. Q. Mai , J. Mater. Chem. A 2015, 3, 18839.

[70] G. Q. Shao , L. Gan , Y. Ma , H. Q. Li , T. Y. Zhai , J. Mater. Chem. A 2015, 3, 11253.

[71] W. T. Kim , B. K. Min , H. C. Choi , Y. J. Lee , Y. U. Jeong , J. Electrochem. Soc. 2014, 161, A1302.

[72] H. C. Liu , P. Hu , Q. Yu , Z. H. Liu , T. Zhu , W. Luo , L. Zhou , L. Q. Mai , ACS Appl. Mater. Interfaces 2018, 10, 23938. 29943974 10.1021/acsami.8b08483

[73] Y. Yang , J. Q. Li , D. Q. Chen , J. B. Zhao , J. Electrochem. Soc. 2017, 164, A6001.

[74] P. Tartaj , J. M. Amarilla , M. B. Vazquez‐Santos , Chem. Mater. 2016, 28, 986.

[75] C. S. Li , Y. Sun , X. G. Ma , W. X. Yao , Y. W. Hu , Y. D. Wang , Y. Z. Wang , RSC Adv. 2014, 4, 9737.

[76] Y. Sun , C. S. Li , L. N. Wang , Y. Z. Wang , X. Ma , P. J. Ma , M. Y. Song , RSC Adv. 2012, 2, 8110.

[77] Y. Sun , C. S. Li , Q. R. Yang , S. L. Chou , H. K. Liu , Electrochim. Acta 2016, 205, 62.

[78] K. T. Nam , D. W. Kim , P. J. Yoo , C. Y. Chiang , N. Meethong , P. T. Hammond , Y. M. Chiang , A. M. Belcher , Science 2006, 312, 885. 16601154 10.1126/science.1122716

[79] J. Xu , P. Liang , D. M. Zhang , C. Y. Pei , Z. P. Zhang , S. Y. Yang , S. B. Ni , J. Mater. Chem. A 2021, 9, 17270.

[80] L. M. Zhu , Z. Li , G. C. Ding , L. L. Xie , Y. X. Miao , X. Y. Cao , J. Mater. Sci. Technol. 2021, 89, 68.

