# Supplementary material for: Novel Li3VO4 Nanostructures Grown in Highly Efficient Microwave Irradiation Strategy and Their In‐Situ Lithium Storage Mechanism
Source: Adv Sci (Weinh). 2021 Nov 21;9(3):2103493. doi: 10.1002/advs.202103493 (PMC8787407; doi:10.1002/advs.202103493)
Supplement: Supplementary file 1 — Supporting Information [file ADVS-9-2103493-s002.pdf]

WILEY-VCH

ADVANCED SCIENCE
Open Access

Supporting Information

for *Adv. Sci.*, DOI: 10.1002/advs.202103493

Novel Li_3VO_4 Nanostructures Grown in High–efficient Microwave Irradiation Strategy and Their *In–situ* Lithium Storage Mechanism

Yan Sun, Chunsheng Li,* Chen Yang, Guoliang Dai, Lin Li, Zhe Hu, Didi Wang, Yaru Liang, Yuanliang Li, Yunxiao Wang, Yanfei Xu, Huakun Liu, Shulei Chou,* Zhu Zhu, Miaomiao Wang, Jiahao Zhu*

Supporting Information

Novel Li_3VO_4 Nanostructures Grown in High−efficient Microwave Irradiation Strategy and Their *In−situ* Lithium Storage Mechanism

Yan Sun, Chunsheng Li,* Chen Yang, Guoliang Dai, Lin Li, Zhe Hu, Didi Wang, Yaru Liang, Yuanliang Li, Yunxiao Wang, Yanfei Xu, Huakun Liu, Shulei Chou,* Zhu Zhu, Miaomiao Wang, Jiahao Zhu*

Prof. Y. Sun, Prof. C. S. Li, Prof. G. L. Dai, C. Yang, D. D. Wang, Z. Zhu, M. M. Wang, J. H. Zhu
School of Chemistry and Life Sciences
Suzhou University of Science and Technology
Suzhou City, Jiangsu Province 215009 (P.R. China)
E-mail: lichsheng@163.com
Dr. L. Li, Dr. Z. Hu, Prof. S. L. Chou
Institute for Carbon Neutralization
College of Chemistry and Materials Engineering
Wenzhou University
Wenzhou, Zhejiang 325035 (P.R. China)
E-mail: chou@wzu.edu.cn
Dr. Y. R. Liang, Dr.Y. X. Wang, Dr. Y. F. Xu, Prof. H. K. Liu
Institute for Superconducting and Electronic Materials
University of Wollongong
Wollongong, NSW 2522 (Australia)
Prof. C. S. Li,
Xi'an Key Laboratory of Advanced Photo-electronics Materials and Energy Conversion Device
School of Science
Xijing University, Xi'an, 710123 (P.R. China)
Prof. Y. L. Li
Hebei Provincial Key Laboratory of Inorganic Nonmetallic Materials
Key Laboratory of Environment Functional Materials of Tangshan City
College of Materials Science and Engineering
North China University of Science and Technology, Tangshan City, Hebei Province, 063210 (P.R. China)

1. The microwave irradiation equipment

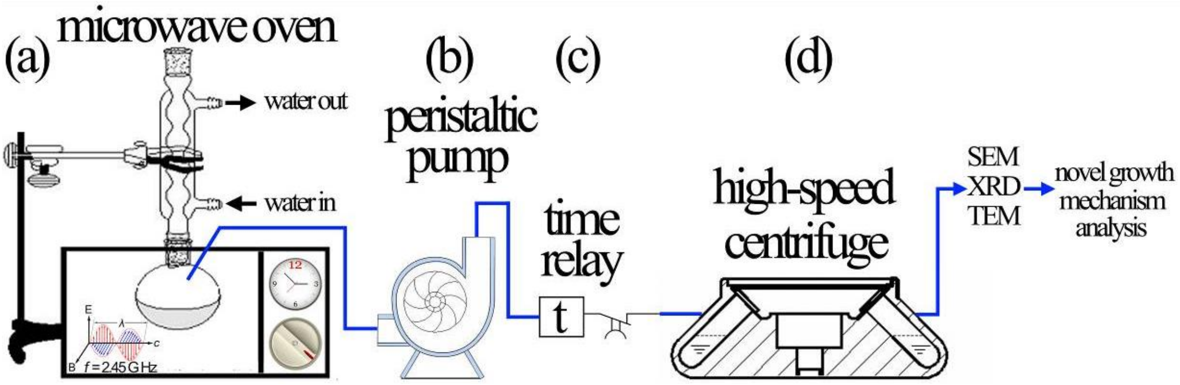

Figure S1. Schematic diagram of microwave irradiation setup: a) A modified microwave oven (Midea PJ21C-AU, frequency: 2.45 GHz); b) The peristaltic pump controls the flow rate of Li_3VO_4 suspension liquid from the microwave oven at continuous time-spans; c) The time relay is a time-controlled switch to manipulate the on and off of the peristaltic pump; and d) The high-speed centrifuge separates the Li_3VO_4 nanoproducts from the suspension liquid, which can further investigate the novel growth mechanism.

2. The experimental parameters and corresponding XRD results

Table S1. Main topologies, XRD data of Li_3VO_4 nanomaterials as a function of corresponding experimental conditions

Sample	Method	Experimental conditions	Morphology	Phase
**1**	Microwave irradiation route	In the experiment, 0.4096 g of V_2O_5 yellow powders were dispersed in 5.0 mL distilled water. While, 5.6660 g of LiOH·H_2O and 0.1457 g of CTAB were dissolved in 18.5 mL distilled water to generate a homogeneous solution using strong magnetic stirring for 10 min. Subsequently, the two solutions are thoroughly mixed in a microwave quartz vessel and the resulting mixture was irradiated on modified microwave equipment (Midea PJ21C-AU) for 1 min. The highly microwave responsive Li_3VO_4 can be rapidly heated to enhance the kinetic process. The collected sample was rinsed with distilled water for several times to remove residual organic compounds and dried at 70 °C for 24 h.	**Hollow nanospheres** (diameter: 1.0-1.5 μm, thickness: 80-100 nm)	Li_3VO_4 JCPDS Card No. 24-667
**2**	Microwave irradiation route	LiOH·H_2O (4.6745 g) and CTAB (0.1457 g) were dissolved in distilled water (18.4 mL). The resulting solution was poured into a V_2O_5 suspension, which contained 0.1126 g V_2O_5 and 5.0 mL distilled water. A microwave reaction for Li_3VO_4 was conducted by irradiating the above precursors in the modified microwave equipment (Midea PJ21C-AU) for 1 h. The collecting procedure of nanomaterials was similar to that used to samples 1.	**Nanoflowers** (diameter: 1.0-2.1 μm, thickness: 25-50 nm)	Li_3VO_4 JCPDS Card No. 24-667
**3**	Microwave irradiation route	0.9444 g LiOH·H_2O and 0.1169 g EDTA are dissolved in 20 mL distilled water. 0.3413g V_2O_5 bulks were dispersed in 5.0 mL by ultrasonication for 0.5 h at 30 °C. After heating the mixture of the two solutions in modified microwave equipment (Midea PJ21C-AU) for 0.5 h, hollow nanocubes are obtained. The product was washed by the same procedure applied for the collection of Samples 1-2.	**Hollow nanocubes** (diameter: 2.0-3.0 μm, thickness: 100-320 nm)	Li_3VO_4 JCPDS Card No. 24-667
**4**	The combination of solid-state method and microwave irradiation	Li_3VO_4 nanowires are synthesized by a facile solid-state reaction and subsequent microwave irradiation route. Firstly, for the solid-state reaction, stoichiometric amounts of Li_2CO_3 (99.9%) and V_2O_5 (99.9%) were thoroughly mixed and pressed at 10 MPa for 10 min, and precalcined at 550 °C for 3 h in air, followed by heating at 900 °C for 5 h in air. Secondly, microwave irradiation fabrication was performed on a modified microwave refluxing setup (Midea PJ21C-AU) using a constant frequency of 2450	**Nanowires** (diameter: 80-240 nm, length: > 3 μm)	Li_3VO_4 JCPDS Card No. 24-667

route	MHz at fast heating power up to 700 W. The 12 g Li_3VO_4 bulks were suspended in 23.5 mL distilled water and introduced into the microwave chamber for 8 h at 100 °C. After cooling, the fine white powders were collected, centrifuged, washed, and dried at 70 °C for 24 h.

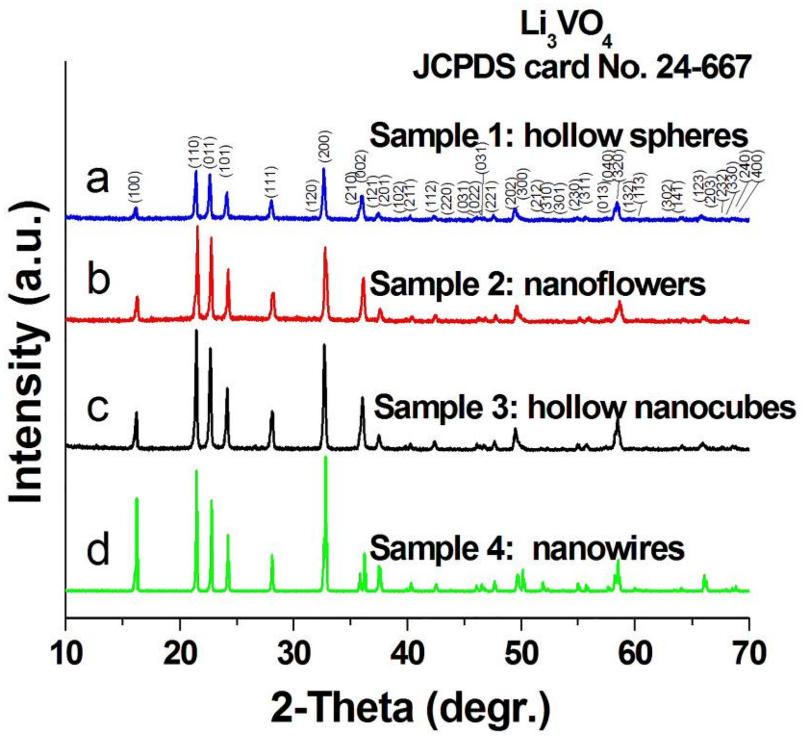

Figure S2. The X-ray diffraction (XRD) patterns of Li_3VO_4 nanostructures with controllable morphologies: a) Sample 1: hollow nanospheres, obtained through high-efficient microwave irradiation method for 1min; b) Sample 2: nanoflowers, gained by microwave irradiation route for 1min; c) Sample 3: hollow nanocubes, achieved by microwave process for 30 min; and d) Sample 4: nanowires, prepared via solid-state reaction subsequent microwave irradiation. The summary of these experimental conditions is described in Table S1.

3. The growth mechanism of various Li_3VO_4 morphologies

3.1 The formation mechanism of Li_3VO_4 hollow spheres in the microwave irradiation route

Table S2. Structural and morphological measurements of Li_3VO_4 hollow nanospheres via a high-efficient microwave irradiation process at selected reaction time according to the synthesizing parameters of Sample 1 in Table S1

Sample	Method	c(V_2O_5) (mmol)	*c*($LiOH·H_2O$) (mmol)	Li/V ratio	*c*(CTAB) (mmol)	Time (s)	Main morphology	Phase
**5**	Microwave irradiation route	2.25	135.09	60:1	0.40	2s	Mesocubes assembled by nanosheets (diameter: 2-3 μm, thickness: 5 nm)	Li_3VO_4 JCPDS Card No. 24-667
**6**	Microwave irradiation route	2.25	135.09	60:1	0.40	10s	Nanospheres (diameter: 0.9-1.5 μm, thickness: 8-15 nm)	Li_3VO_4 JCPDS Card No. 24-667
**7**	Microwave irradiation route	2.25	135.09	60:1	0.40	20s	Nanospheres and hollow nanospheres (diameter: 1.2-1.8 μm, thickness: 12 nm)	Li_3VO_4 JCPDS Card No. 24-667
**8**	Microwave irradiation route	2.25	135.09	60:1	0.40	40s	Hollow nanospheres with nanoballs in the core (diameter: 2.1-6.8 μm, thickness: 100-180 nm)	Li_3VO_4 JCPDS Card No. 24-667
**9**	Microwave irradiation route	2.25	135.09	60:1	0.40	60s	Hollow nanospheres (diameter: 1.2-2.5 nm, wall thickness: 54 nm)	Li_3VO_4 JCPDS Card No. 24-667

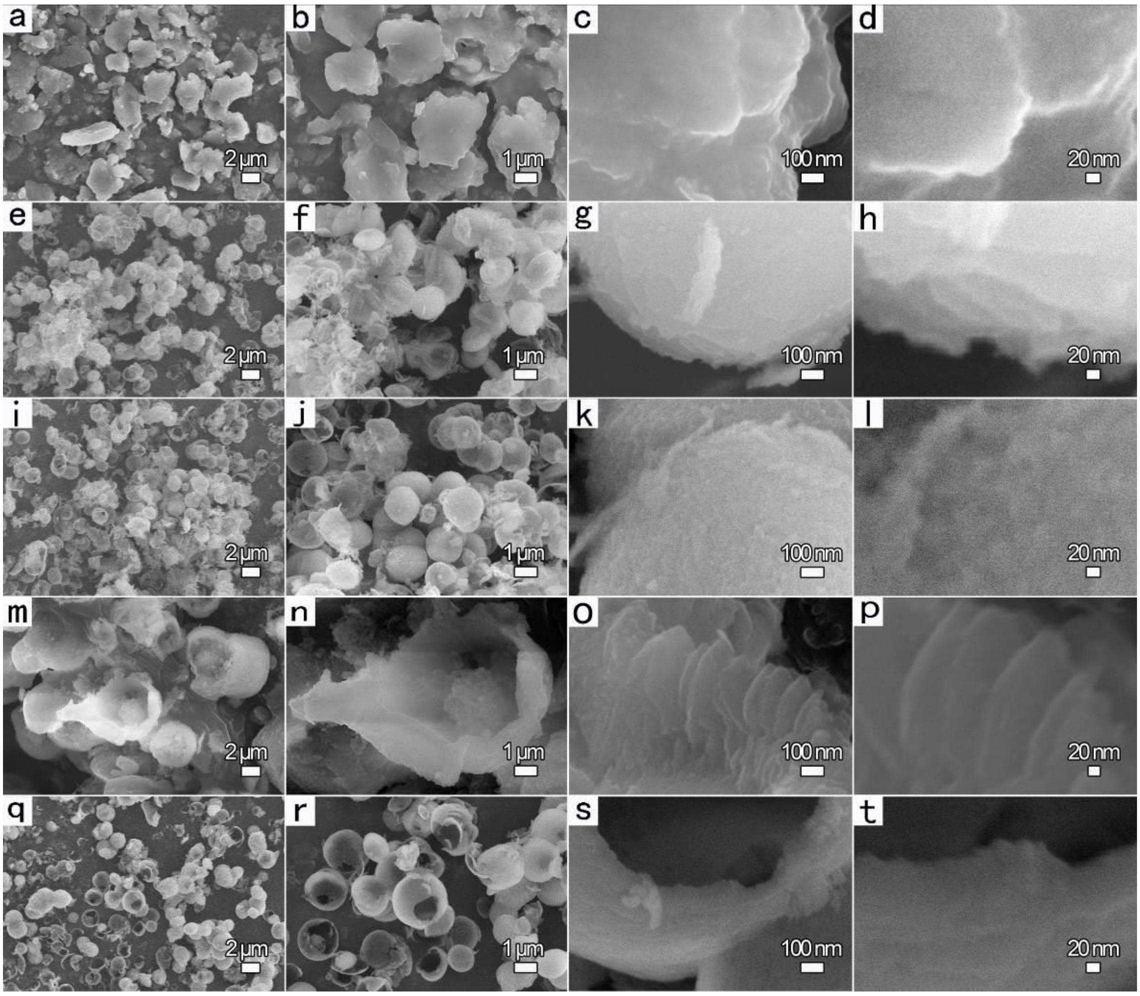

Figure S3. The shape evolution of Li_3VO_4 hollow nanospheres: Low-magnification SEM images of as-synthesized Li_3VO_4 hollow nanospheres with different irradiation time (see Table S2): a-d) Sample 5, 2s, mesocubes assembled by nanosheets; e-h) Sample 6, 10s, nanospheres with a diameter of 0.9-1.5 µm; i-l) Sample 7, 20s, Nanospheres and hollow nanospheres with a size of 1.2-1.8 µm; m-p) Sample 8, 40s, hollow nanospheres with nanoballs in the core; and q-t) Sample 9, 60s, hollow nanospheres with 1.2-2.5 nm in diameter. These SEM images demonstrate the predominance of hollow spheres with a high yield, displaying the novel growth mechanism of the unique nanostructure.

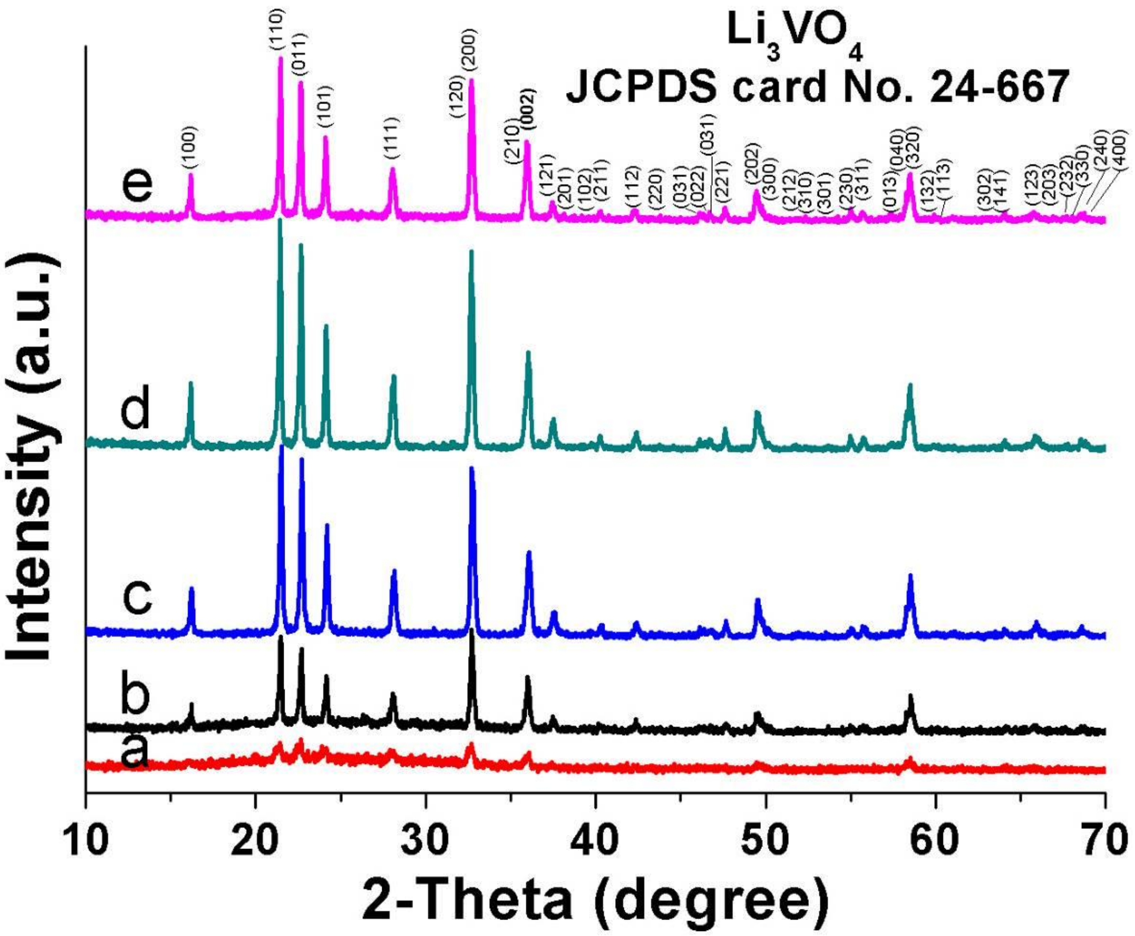

Figure S4. Typical XRD patterns of Li_3VO_4 hollow nanospheres generated after the microwave irradiation assisted-CTAB route at 100 °C for different time-spans: a) Sample 5, 2s; b) Sample 6, 10s; c) Sample 7, 20s; d) Sample 8, 40s; and e) Sample 9, 60s.

3.2 The growth mechanism of Li_3VO_4 hollow spheres in the microwave irradiation route

Table S3. Synthesizing conditions and their corresponding phase and topology of Li_3VO_4 nanoflowers

Sample	Method	c(V_2O_5) (mmol)	*c*($LiOH·H_2O$) (mmol)	Li/V ratio	*c*(CTAB) (mmol)	Time (s)	Main morphology	Phase
**10**	Microwave irradiation route	0.62	111.45	180:1	0.40	2s	Nanoflowers (diameter: 0.4-0.7 µm, thickness: 10 nm)	The mixture of Main Li_3VO_4 (JCPDS Card No. 24-667) and $LiVO_3$ (70-1545)
**11**	Microwave irradiation route	0.62	111.45	180:1	0.40	10s	Nanoflowers (diameter: 0.5-1.0 µm, thickness: 8 nm)	The mixture of Main Li_3VO_4 (JCPDS Card No. 24-667) and $LiVO_3$ (70-1545)
**12**	Microwave irradiation route	0.62	111.45	180:1	0.40	20s	Nanoflowers (diameter: 0.3-0.7 µm, thickness: 12 nm)	Main phase of Li_3VO_4 (JCPDS Card No. 24-667) and trace $LiVO_3$ (70-1545)
**13**	Microwave irradiation route	0.62	111.45	180:1	0.40	40s	Nanoflowers (diameter: 1.0-1.5 µm, thickness: 10-50 nm)	Main phase of Li_3VO_4 JCPDS Card No. 24-667
**14**	Microwave irradiation route	0.62	111.45	180:1	0.40	60s	Nanoflowers (diameter: 1.2-2.0 µm, thickness: 12 nm)	High pure Li_3VO_4 (JCPDS Card No. 24-667)

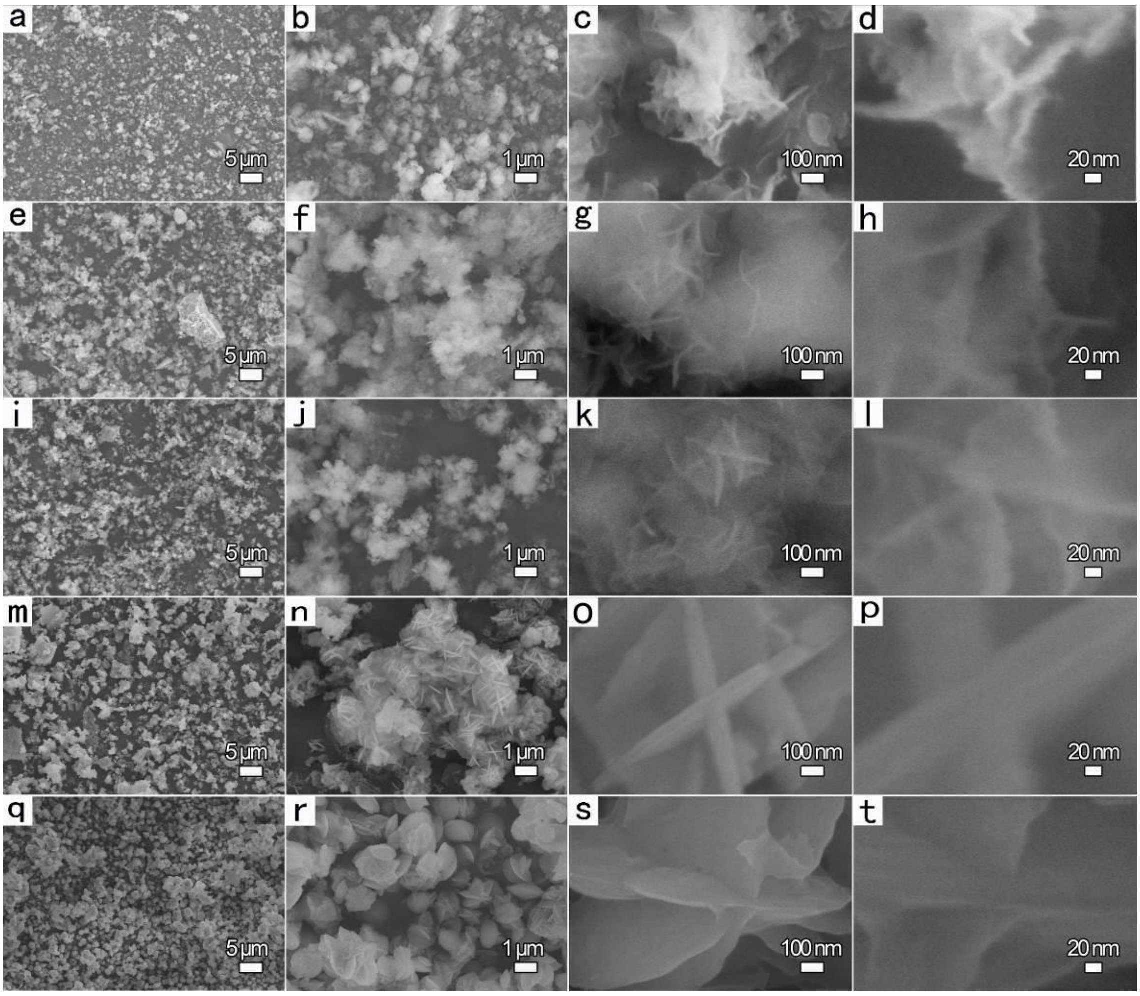

Figure S5. The growth process recorded from homogeneous Li_3VO_4 nanoflowers via the microwave irradiation route: SEM images with different magnifications of as-prepared samples containing a high quantity of Li_3VO_4 nanoflowers with different sizes at typical fabricating stages (see Table S3): a-d) Sample 10, 2s; e-h) Sample 11, 10s; i-l) Sample 12, 20s; m-p) Sample 13, 40s; and q-t) Sample 14, 60s.

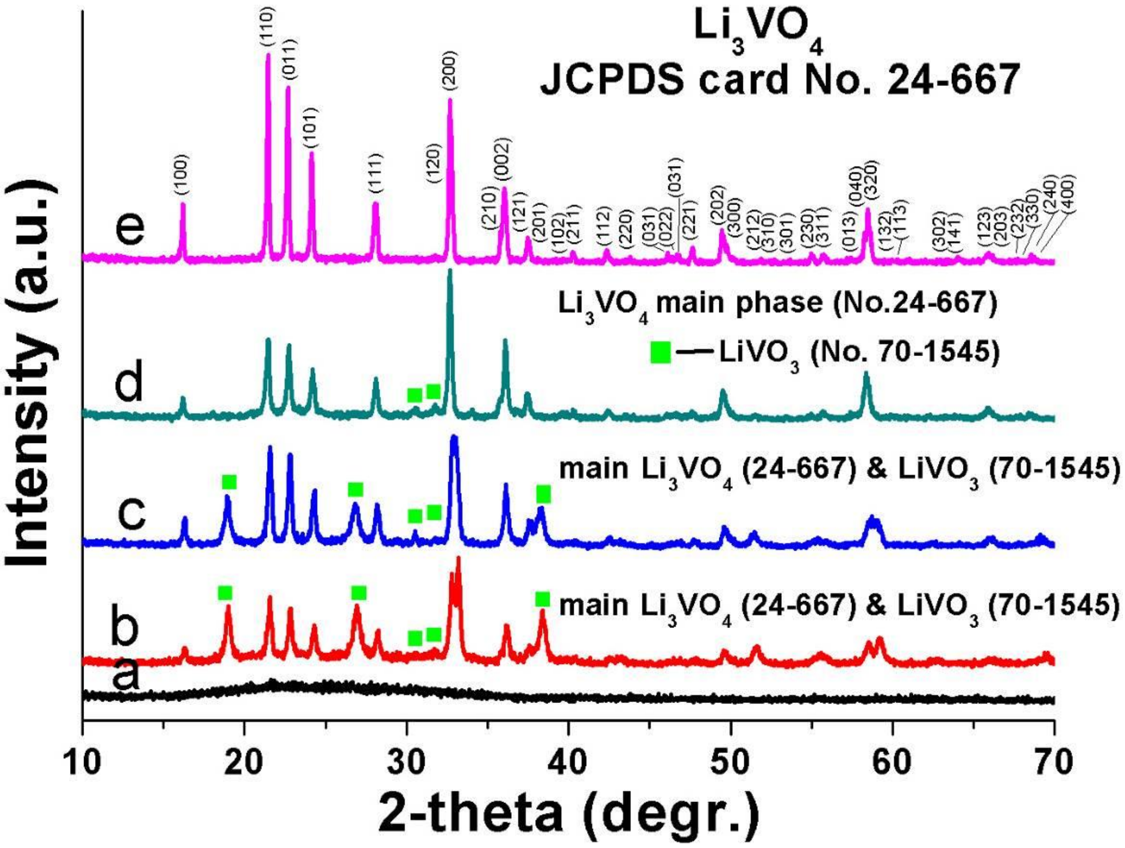

Figure S6. XRD characterization of Li_3VO_4 3D nanoflowers (Sample 10-14) architecture constructed from ultrathin nanosheets at various time: a) Sample 10, 2s; b) Sample 11, 10s; c) Sample 12, 20s; d) Sample 13, 40s; and e) Sample 14, 60s. The green squares "■" denote the JCPDS card No.70-1545 file attributed to $LiVO_3$.

3.3 The growth mechanism of Li_3VO_4 hollow nanocubes in the microwave irradiation method

Table S4. Microstructural evaluation of Li_3VO_4 hollow nanocubes through microwave irradiation assisted EDTA method

Sample	Method	c(V_2O_5) (mmol)	*c*($LiOH{\cdot}H_2O$) (mmol)	Li/V ratio	*c*(EDTA) (mmol)	Time (min)	Main morphology	Phase
**15**	Microwave irradiation route	1.88	22.52	20:1	0.40	1	Square-like nanosheets (side length: 0.7-2.0 μm)	The mixture of Main Li_3VO_4 (JCPDS Card No. 24-667) and $LiVO_3$ (70-1545)
**16**	Microwave irradiation route	1.88	22.52	20:1	0.40	3	Nanocubes (side length: 1.5-2.6 μm)	The mixture of Main Li_3VO_4 (JCPDS Card No. 24-667)
**17**	Microwave irradiation route	1.88	22.52	20:1	0.40	5	Nanocubes with hollows in the center (side length: 1.4-1.8 μm)	The mixture of Main Li_3VO_4 (JCPDS Card No. 24-667)
**18**	Microwave irradiation route	1.88	22.52	20:1	0.40	15	Nanocubes with hollows in the center (side length: 1.2-2.7 μm)	High pure Li_3VO_4 (JCPDS Card No. 24-667)
**19**	Microwave irradiation route	1.88	22.52	20:1	0.40	20	Hollow nanocubes (side length: 2.2-3.3μm, the nanosheets of walls thickness: 25-40 nm)	High pure Li_3VO_4 (JCPDS Card No. 24-667)

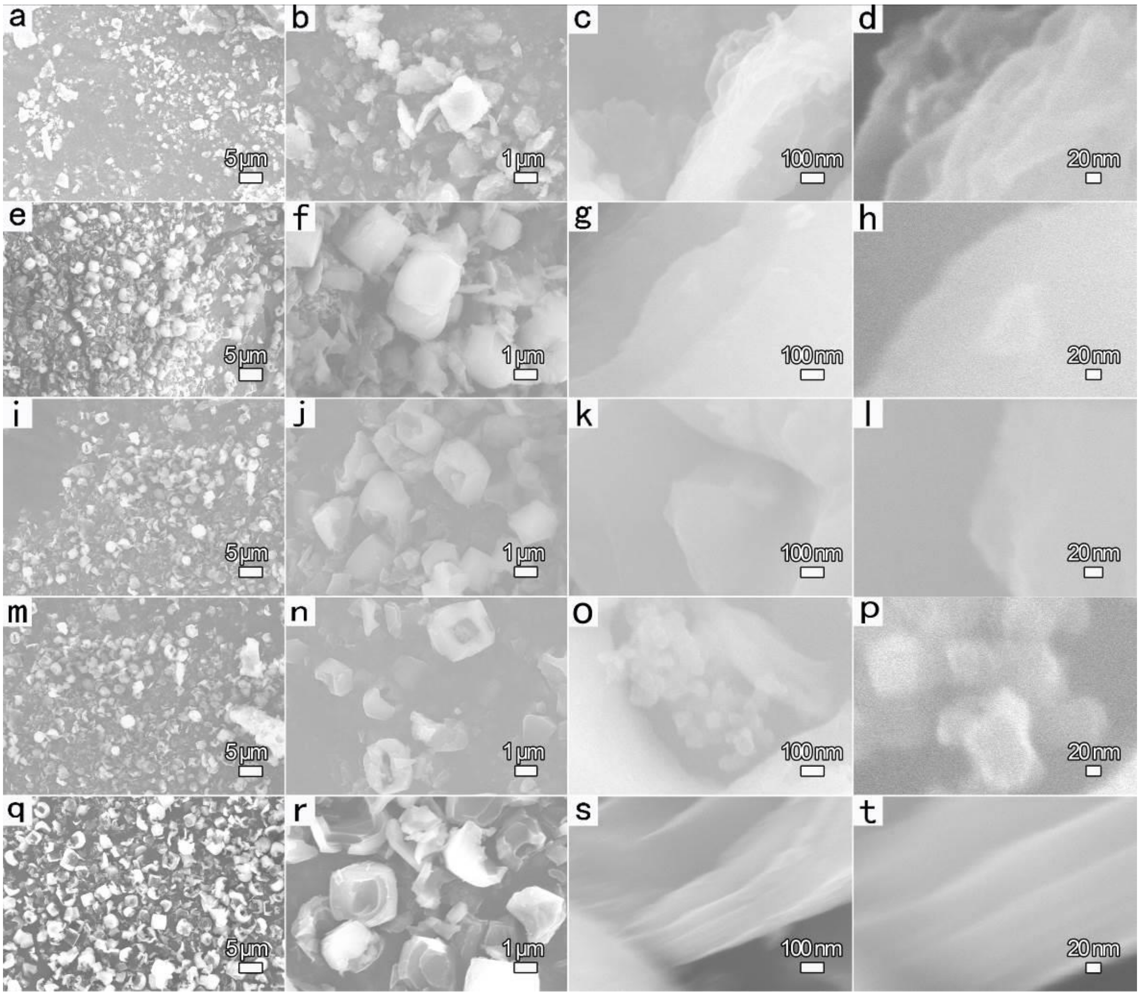

Figure S7. The shape monitor of Li_3VO_4 hollow nanocubes at different dwell time. SEM images of Li_3VO_4 cubes prepared by a microwave radiation-assisted EDTA route as described in Table S4, clearly showing the dissolution-recrystallization process from the core of the nanocubes: a-d) Sample 15, 1 min, square-like nanosheets; e-h) Sample 16, 3 min, nanocubes; i-l) Sample 17, 5 min, nanocubes with hollows in the center; m-p) Sample 18, 15 min, nanocubes with hollows in the center; and q-t) Sample 19, 20 min, hollow nanocubes.

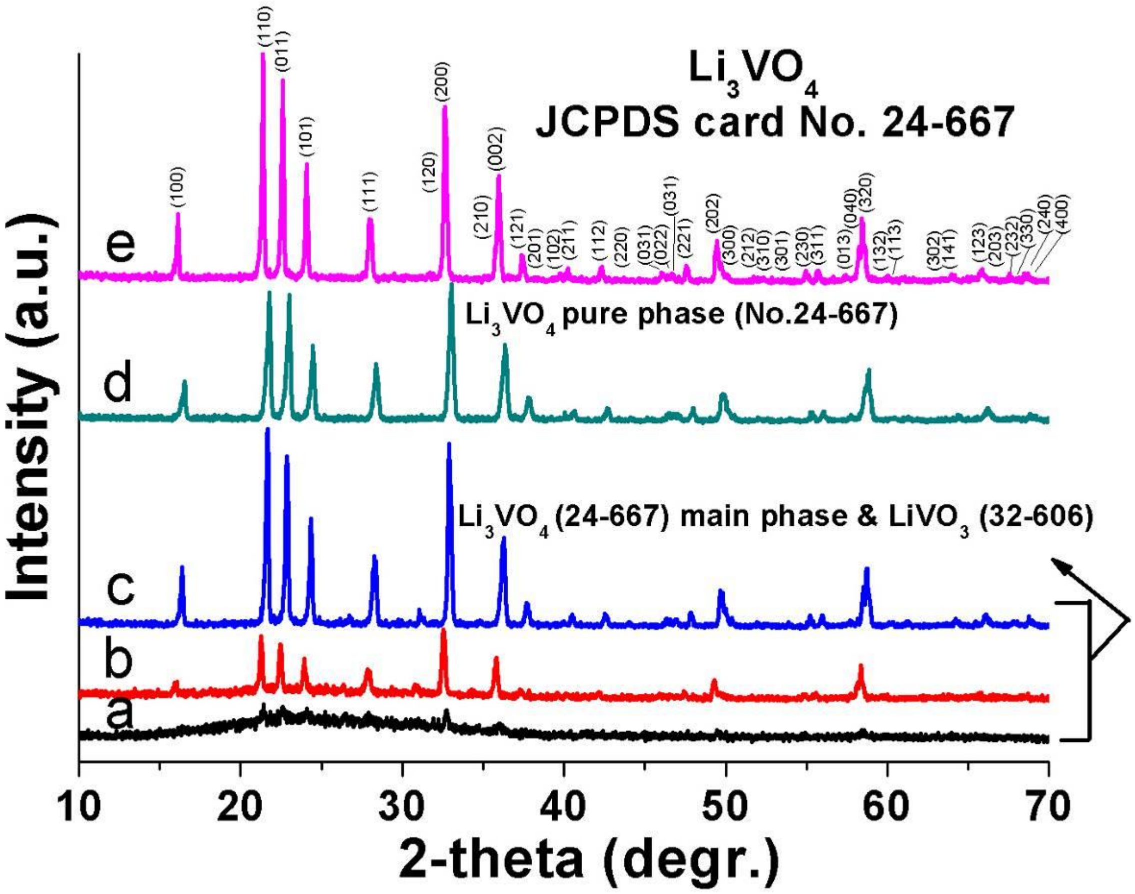

Figure S8. The evolution of crystalline structure for as-grown Li_3VO_4 hollow cubes with the absence of EDTA in microwave irradiation at a certain heating time: a-d) Sample 15, 1min; e-h) Sample 16, 3min; i-l) Sample 17, 5min; m-p) Sample 18, 15min; and q-t) Sample 19, 20min.

3.4 The growth mechanism of Li_3VO_4 nanowires

Table S5. The growth mechanism of Li_3VO_4 nanowires via the combination of the solid state method and microwave irradiation process at various time intervals

Sample	Method	Solid-state method condition	Microwave irradiation time (h)	Main morphology	Phase
**20**	Solid-state reaction and subsequent microwave irradiation route	n(Li_2CO_3:V_2O_5)= 3:1, heated at 550 °C for 3 h in air, followed by calcined at 900 °C for 5 h in air	1	Microscale bulks (size: 1.3-10.0 μm)	High pure Li_3VO_4 (JCPDS Card No. 24-667)
**21**	Solid-state reaction and subsequent microwave irradiation route	n(Li_2CO_3:V_2O_5)= 3:1, heated at 550 °C for 3 h in air, followed by calcined at 900 °C for 5 h in air	2	Nanowires irradiated from microbulks (diameter: 50-220 nm, length: > 5 μm)	High pure Li_3VO_4 (JCPDS Card No. 24-667)
**22**	Solid-state reaction and subsequent microwave irradiation route	n(Li_2CO_3:V_2O_5)= 3:1, heated at 550 °C for 3 h in air, followed by calcined at 900 °C for 5 h in air	4	Mesospheres assembled from numerous nanoneedles (diameter: 60-270 nm, length: > 2 μm)	High pure Li_3VO_4 (JCPDS Card No. 24-667)
**23**	Solid-state reaction and subsequent microwave irradiation route	n(Li_2CO_3:V_2O_5)= 3:1, heated at 550 °C for 3 h in air, followed by calcined at 900 °C for 5 h in air	8	Uniform nanowires (diameter: 100-300 nm, length: > 1.5 μm)	High pure Li_3VO_4 (JCPDS Card No. 24-667)

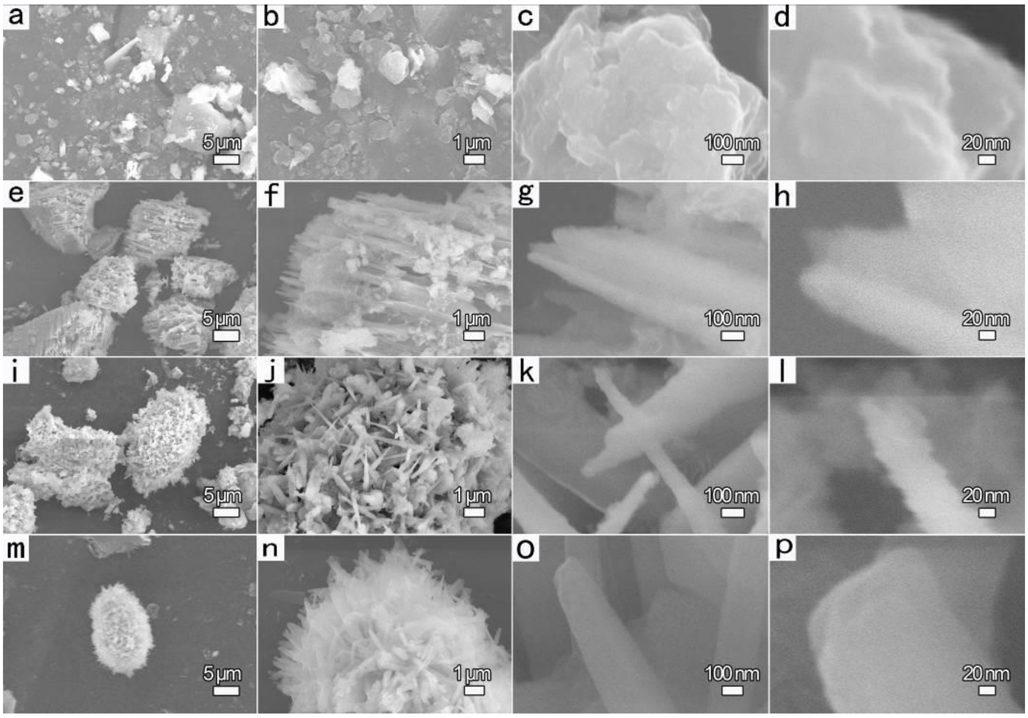

Figure S9. The morphological change of Li_3VO_4 nanowires. Representative SEM images of Li_3VO_4 products fabricated through the combination of Solid-state reaction and microwave irradiation method with the same concentrations of raw materials and heating temperature of 100 °C but continuously altering the reaction time intervals as listed in Table S5: a-d) Sample 20, microscale bulks with a size of 1.3-10.0 μm, 1h; e-h) Sample 21, nanowires irradiated from microbulks with 50-220 nm in diameter and 5 μm in length, 2h; i-l) Sample 22,mesospheres assembled from numerous nanoneedles (diameter: 60-270 nm), 4h; and m-p) Sample 23, uniform nanowires with diameter in the range of 100-300 nm and length in at least 1.5 μm, 8h.

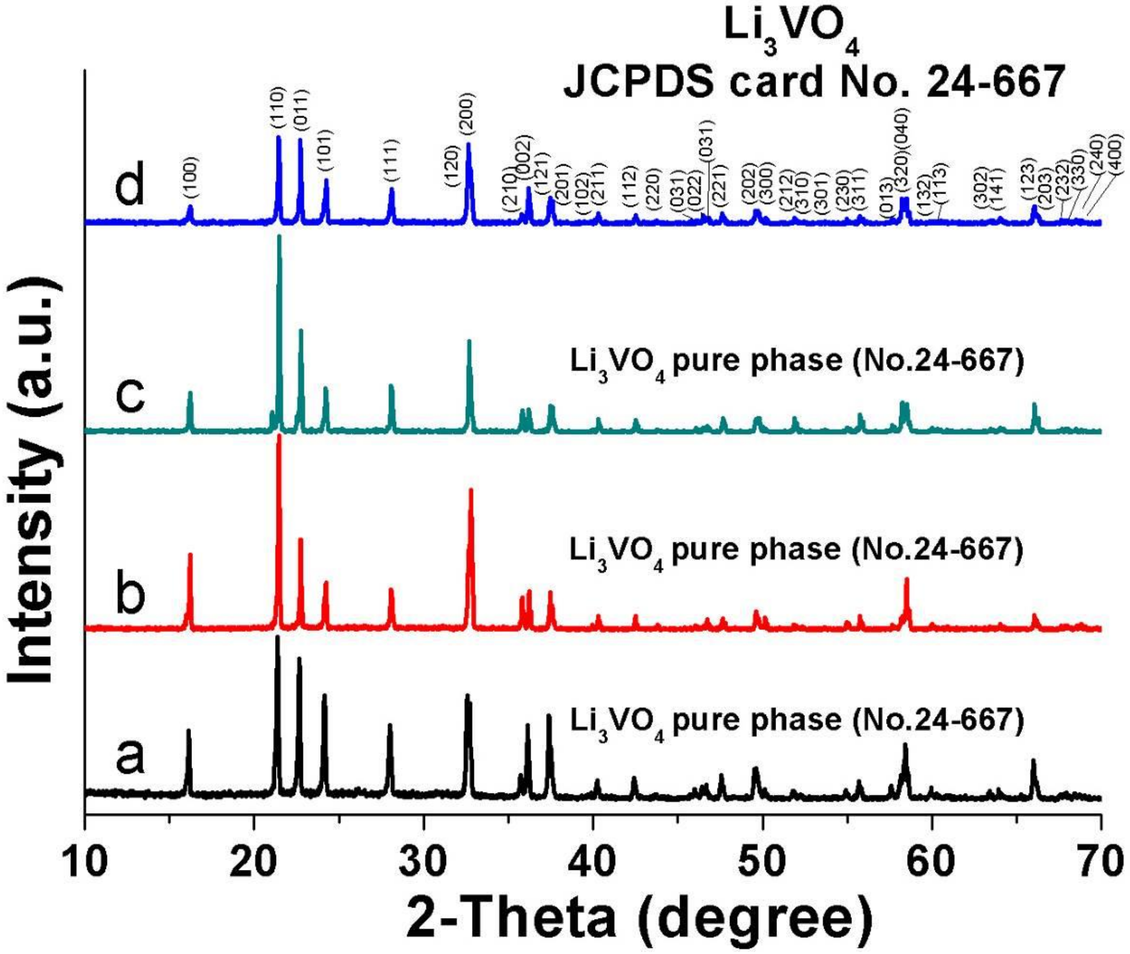

Figure S10. XRD patterns of Li_3VO_4 gained through the same solid state method but subsequent microwave irradiation process at different reaction intervals: a) Sample 20, 1 h; b) Sample 21, 2 h; c) Sample 22, 4 h; and d) Sample 23, 8 h.

4. Electrochemical Characterization:

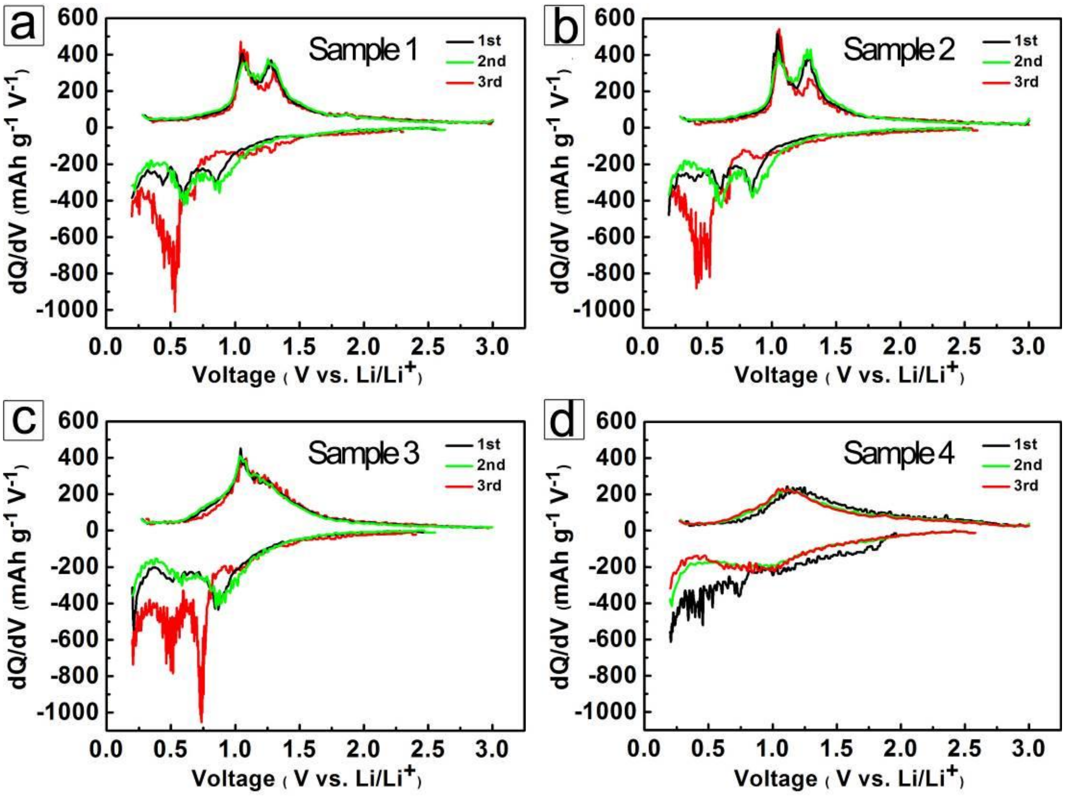

Figure S11. Cyclic voltammetry (CV) of Li_3VO_4 electrodes at a scan rate of 0.05 $mV{\cdot}s^{-1}$ ranging from 0.20 to 3.00 V: a) Sample 1: hollow nanospheres; b) Sample 2: nanoflowers; c) Sample 3: hollow nanocubes; and d) Sample 4: nanowires.

Movie S1. Video recording the morphological evolution in *in−situ* TEM characterization of a single Li_3VO_4 hollow nanosphere before and after (de)lithation process

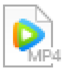

4 Movie S1 naosphere.mp4
